# Conforming to partnership values: a qualitative case study of public–private mix for TB control in Ghana

**DOI:** 10.3402/gha.v9.28000

**Published:** 2016-01-05

**Authors:** Joshua Amo-Adjei

**Affiliations:** Department of Population and Health, Faculty of Social Sciences, College of Humanities and Legal Studies, University of Cape Coast, Cape Coast, Ghana

**Keywords:** Public–private mix, beneficence, non-maleficence, equity, autonomy

## Abstract

**Background:**

Public–private mix (PPM) can supplement public sector initiatives, including public health. As National Tuberculosis Control Programmes around the world embrace PPM, conforming to the four key principles of partnership values of beneficence, non-maleficence, autonomy, and equity as espoused by the World Health Organization can provide a useful framework to guide successful implementation.

**Design:**

This is a qualitative case study of PPM in tuberculosis (TB) control, which utilised a purposive sample of 30 key stakeholders involved in TB control in Ghana. Respondents comprised an equal number of respondents from both the public and private sectors. Semi-structured in-depth interviews (IDI) were conducted with respondents. Data emanating from the IDIs were analysed deductively.

**Results:**

Although the respondents’ perceptions about beneficence were unanimous, their views about non-maleficence, autonomy, and equity appeared incongruous with the underlying meanings of the PPM values. Underlying the unfavourable perceptions were disruptions in funding, project implementers’ failure to follow-up on promised incentives, and private providers lost interest. This was perceived to have negatively affected the smooth implementation of PPM in the country.

**Conclusions:**

Going forward, it is imperative that future partnerships are built around utilitarian principles and also adhere to the dictates of agreements, whether they are ‘soft’ or standard contracts.

## Introduction

The public health sector of several countries has embraced public–private mix (PPM) for the purposes of improving health through product development and access to critical care (1–4). In most parts of the world, publicly owned and managed health services face challenges. In low- and middle-income countries, health services are characterised by inadequate infrastructure and frequent shortages of supplies, which result in low quality of care (4, 5). The large presence of the private sector (6) has been the key premise for including them in the delivery of critical health services such as DOTS.

A recent systematic review (7) showed that the private sector fared better on some dimensions of quality of health delivery (e.g. timeliness and hospitality towards patients), although the evidence on efficiency and accountability were inconclusive. In tuberculosis (TB) control, several studies (8–18) have confirmed the importance of the private sector in accelerating positive treatment outcomes. Nevertheless, there are some sceptics (19–22), who have questioned whether private facilities are really making any useful contribution to TB treatment. Olson et al. (22) have partly attributed the persistence of drug-resistant TB to the high involvement of private facilities in TB treatment in some parts of India, and after two decades of PPM in India, Udwadia et al. (21) are of the view that there is really nothing to show.

In 1999, the World Health Organization (WHO) (23) proposed four important values to guide effective partnership between public and private health services provision. These are beneficence (partnership should lead to public health gain), non-maleficence (must lead to public good), autonomy (partnership should not undermine each partner’s autonomy), and equity (benefits should be distributed to those most in need). In 2003, Ghana’s National Tuberculosis Control Programmes (NTP) rolled out PPM in two regions – Ashanti and Greater Accra, with the objective of scaling up to other parts of the country. Complete scale-up is yet to be achieved in the country (24). The literature on TB in Ghana and other developing countries is increasing but other salient issues such as PPM have not received much empirical discourse. A very recent review of the general management and public health literature on PPM revealed that about 63% of research evidence on the subject emanate from the United States and United Kingdom (25). In respect of PPM for TB control, much of the research has come from high-burden countries, such as India, Pakistan, South Africa, and China (8–22). Given this paucity of empirical studies on PPM in general, and in particular on TB, this study is intended to explore the experiences of frontline health personnel with the implementation of PPM for TB control in the country. Apart from the geographical coverage, barely any of the previous studies is conceptually driven. Thus, by borrowing the WHO partnership framework, this paper sought to examine the perspectives of key stakeholders on how partnership values/ethics evolved over the period of implementation and what lessons can be learnt for future interventions in scaling up in Ghana and maybe, in other developing countries.

## Methods

### Research setting

The NTP 2009–2013 strategic plan purposed to take advantage of the huge presence of the private sector in Ghana’s health delivery to improve TB case detection and access to TB care services through inclusive service provision (24). As of 2013, there were 1,500 health facilities providing TB-related services in the country comprising 130 private health facilities and 28 private laboratories involved in TB diagnosis and treatment throughout the country (26).

The PPM model operating in the country is similar to soft or relational contracting, which is a ‘mutual agreement between the collaborative partners about the general terms of collaboration’ (p. 876) with limited financial considerations unlike standard contracts where indemnities exist; parties may opt out depending on exigencies (27). In Ghana, a Memorandum of Understanding (MoU) was signed between the public sector and every enlisted private facility. Private facilities reserved the right to participate or not in the TB control activities.

With the support of the Global Fund to Fight AIDS, TB, and Malaria (GFATM), financial provisions were made for patients, healthcare workers, and health facilities: 50% of the enabler’s package^1^ (EnP) went to TB clients; 30% to the healthcare workers involved in TB care, and 20% to the facility (50:30:20) (28). The amount of money dedicated to was not constant; it varied depending on the funding available but it ranged from $40 to $100. Indeed, the overall funding scheme of the NTP from both external and internal sources has been unstable. For instance, in 2013, 45% of the NTP total budget ($38,000,000) was funded ($17,100,000), and of the proportion funded, 84% ($14,440,000) was received from external sources, with the remaining funded locally (29). In the last few years, the contributions of the private sector to TB treatment outcomes were 13.4, 11.2, 4.2, and 5.6% for 2010, 2011, 2012, and 2014, respectively.

### Data collection

This study targeted key personnel in TB control at the national, regional, and metropolitan/municipal/district levels; and private service providers in the Greater Accra and Asante regions. These two sites were purposively chosen because PPM was piloted in these two areas and are currently areas with relatively the highest number of private sector participation. Thirty purposively selected respondents, 15 from the public and 15 from the private sectors were interviewed for the study and this was informed by an *a-priori* logic that in partnerships, partners must be seen as co-equals.

A similar semi-structured interview guide was used for both public and private facility respondents but with some minor variations in questioning. With the consent of the respondents, all the interviews were tape-recorded, transcribed, and edited. All the interviews were conducted in English because the respondents were all literate in this language.

We conducted in-depth interviews in a very flexible approach in order to allow interviewees to adequately express their views on the key constructs of partnership ethics/values. The data collection and transcription were done concurrently to allow for clarifications of emerging issues with participants. Despite the similarities in the questions, questioning varied slightly depending on whether the respondent was from the private or the public sector, given the differences in role. For instance, a respondent from the private sector would be asked their motivations for accepting the PPM model, whereas a public sector respondent attached to the programme was asked reasons for initiating PPM. Whereas the former brought up the initiative, the latter was either to accept or reject. Respondents were asked about perceptions on benefits, personnel, logistical constraints, training and development, monitoring, and evaluation. The interview guide was pretested and interviews were conducted in English between March and August 2012. Follow-up interviews with a few of the respondents were conducted between August and October 2014. The aim was to validate and clarify some of the preliminary findings, particularly those from the private sector respondents. The author conducted, audiotaped, and transcribed all the interviews. The University of Cape Coast, Ghana, Institutional Review Board gave ethical approval for the study.

### Data analysis

A deductive content (30) approach was taken to analyse the data. A codebook was developed around beneficence, non-malfeasance, equity, and autonomy. QSR NVivo 9 was used to assist in the coding. To enhance the validity of the coding, three experts in qualitative research reviewed the codes for consistency. Besides, a draft report of the study was evaluated by five of the respondents, selected purposively.

## Findings

The following are the characteristics of the respondents: 5 national programme officers, 5 management members of private facilities, 10 nurses/doctors responsible for TB in the selected private facilities, 2 regional TB coordinators, and 8 district/metropolitan TB coordinators. In the proceeding, we report on the issues that emanated from the respondents with regard to their views about PPM in Ghana’s TB Control Programme.

### Beneficence

Respondents unanimously agreed that PPM was an important addition to the TB control efforts in the country. The general view was that with the inclusion of the private sector in both diagnosis and treatment, the programme had become more visible in the communities. The general view was that PPM DOTS contributed to achieving important milestones by improving access to diagnosis and treatment to many people who could have been missed out because of the limited coverage of the public health sector. Some of the participants expressed their views about the benefits as follows:

Overall, I think the involvement of the private sector was an important innovation. It helped us to increase diagnosis modestly and more highly in terms of treatment outcomes. Because of the difficulties in accessing the few public health facilities, we were able to capture a number of patients; some of whom were allocated to a number of private facilities such as maternity homes. It invariably reduced the travelling time and cost to patients. (District TB coordinator)

In all the partnership has worked well. I also perceive that defaulter rates are low in the private facilities but that may be due to the smaller number of cases they deal with. (National TB programme officer)

I will say it has been good since it is, and continues to help widen the scope of outreach of care to many tuberculosis patients. (Nurse, private hospital)

So far, I think they have made positive input into our tuberculosis control programme. The information I have is that they have been successful; they notify 10–13% of cases and their treatment success is above the national average from public facilities. (National TB programme officer)

I can say we are doing well. This is because the majority of cases we observe are treated successfully except that there are few who default treatment. (Nurse, private hospital)

To some other respondents, by bringing private facilities on board, it was an opportunity for the private sector to increase their client base. The view was that if those in the private sector are able to deliver satisfactory services to TB patients, the likelihood of the cured and satisfied clients sticking to them for their future health needs was certain. Thus, beneficence was seen by a few of the respondents in terms of the potentials for future increases in the volume of clientele.

I see our participation as helpful because in the long-term, these patients are possibly going to return to us for other health needs once we are able to serve them well. It is more akin to investing in our own future. (Medical doctor, private facility)

### Non-maleficence

The non-maleficence principle in public health PPM is expressed in two dimensions – either to the patient or to a partner. Views from the private sector revealed that the partnership did not favour some health facilities. For those within the private sector, their major concern was that funding for TB services was irregular. This comes at the precincts of their inability to charge for TB diagnosis and treatment. Some respondents from the NTP corroborated these views but they also attributed this situation to inadequate funding. The confluence of these situations is a declining participation of private facilities in TB-related activities. One respondent shared the following observation:

The involvement of private facilities in TB control has gone down due to decline in funding. The initiative was started with Global Fund to fight AIDS, TB and Malaria [GFATM] support, and, with the dwindling fortunes from the fund, PPM DOTS has gone down drastically. (Regional TB coordinator)

On perception of failure of the NTP in fulfilling its resource obligations, a respondent asserted as follows:

The genesis of current lackadaisical attitude of private facilities in PPM DOTS is emanating from the fact that most of them were ‘bait’ into TB with financial and materials (e.g. instruments) benefits they stood to gain from enrolling. Now that there no more sufficient fund, some of them have completely withdrawn from TB control (diagnosis and treatment). It is important that private health institutions are made to understand why they need to provide services to TB patients, as it is a public good …. (District TB coordinator)

A few of the respondents held the view that expectations of private providers were not well managed in terms of the tangible and intangible benefits which might accrue from providing TB care. A respondent noted

I feel that we could not manage expectations of private facilities very well. Expectations of private facilities were raised high, as many private providers thought their involvement in TB control was going to result in massive infrastructural changes as well as other benefits to their facilities. When those expectations were not met, most of them have sat back …. (Regional TB coordinator)

Nonetheless, there were some private facilities still providing TB services. To these, their argument was that TB is a threat to public health and therefore provision of services should solely not be measured by tangible benefits. Some respondents noted

… TB care is more of a national service rather than profit-making care because TB treatment is actually free. We do that to save ourselves because TB is an airborne disease so if we contribute to cutting down on the prevalence levels, we save ourselves from high exposure; it will be limited. (Pharmacist, private facility)

The 20% of the EnP for health facilities helped this facility to provide motivated services to TB patients. It is not the case presently but it is just by the benevolence of this hospital’s management (religious-sponsored facility) who provide me with call credit worth $3 as a means of facilitating patient follow-ups. (Nurse, private facility)

### Equity

Two issues that are relevant to equity, which emerged from our interactions, are in respect of perceived inequities in training opportunities or in-service training offered to public and private facilities, as well as the distribution of resources (physical infrastructure/facilities for diagnosis and financial). In terms of training, respondents from private facilities generally lamented about the inadequacies in professional training on TB management. They frequently spoke about the biases associated with in-service training – more emphasis on those in public practice. A respondent expressed the following ‘frustrations’:

I feel that those of us within the private facilities do not receive as much training as those within the public service. Workshops, seminars, and other training programmes are regularly conducted for our colleagues within the public health sector but that is not so with those of us in private institutions. Once a while you are called but in most cases, nobody really remembers us. (Medical doctor, private facility)

I have been here for about three months but I have not had any formal training. The only form of training I have had was the basic rudiments my predecessor gave me. (Nurse, private facility)

In terms of material resources, there were a few respondents who reported occasional cuts in commodities.

There is need for constant supply of diagnosis logistics for us in private practice …. At times, the laboratory personnel complain about erratic supply of reagents. (Nurse, private facility)

At the national programme level, the situation was attributed largely to funding challenges. They were of the view that a greater dependence of the programme donor funds was primarily responsible for such occurrences:

Mainly, the challenge of equitable distribution of resources has a lot to do with funding. Although a number of private facilities consider their involvement in TB activities as part of a national duty. However, some cost has to be borne by the programme. We therefore have to provide these facilities with some funds to meet their overhead cost through the support we receive from GFATM. What it means then is that if we don’t get funds from GFATM, then we cannot support the private facilities. (National programme officer)

### Autonomy

The practice of the programme predetermining a higher proportion of the enablers’ package for individual health workers, which is more than the respective health facilities, was seen as indiscreet/meddling. As expected, management members of private facilities expressed this view more commonly. Their view was that, it was them (the management) and rather not the NTP which should determine how much of the Enabler’s Package went to the staff. The basis of their argument was that because those workers were employees and also worked in their facilities, a higher proportion should have been given to the facility. A respondent noted:

I am concerned about the basis for determining who got what from the enabler’s package. How do you give more to an employee who but for my engagement would not be aware of any TB programme? I don’t think this is fair! (Proprietor, private hospital)

Consequently, this has resulted in situations where health workers in certain private facilities were denied their share of the enabler’s support. A respondent expressed this concern in the following:

… Often, the share of enablers meant for TB coordinators are not given to them because some of the managers feel that the coordinators are their employees who have been paid to do specific jobs. As such, they claim to have prerogative over the use of incoming funds regardless of what workers have done since they pay their salaries … in the end, some private hospitals and clinics deny health workers their share of the enablers’ package. (District TB coordinator)

However, this situation was not universal across all private facilities as there was evidence of particular private hospitals wholly giving the enablers’ package to their DOTS nurses whenever it was available. Interviews with NTP staff also showed that the proposal for the GFATM funding had clearly indicated what these funds would be used for, which made it impossible to divert to other routes.

## Discussion

This study was undertaken to explore the views of frontline staff on PPM for TB control in Ghana on how key partnership values – beneficence, non-maleficence, equity, and autonomy emerge from discussions with key personnel who played varied roles in the implementation of the PPM DOTS. Much as respondents overwhelmingly agreed to the positive effects of PPM in Ghana’s TB control efforts; concerns about non-maleficence, equity, and autonomy somehow affected smooth implementation. No disaggregated data on key treatment outcomes were available for verification, though.

The views of respondents on beneficence can be summed into those accruing to individual patients, participating private health facilities, and the general health system. For individual patients, the cost of transportation (travel time and financial) is reduced by increased access to several options, consistent with findings from previous studies on PPM in TB control (8–18).

Generally, expressions of respondents about non-maleficence were inconsistent with expectations, especially with regard to syndication ethics, which purports that a partner to a partnership must not suffer disproportionately; whether perceived or real. Such perceptions or realties could negatively affect the credibility of partners. More worrying in such instances is the potential it has to discourage prospective partners. Given that private facilities are profit oriented, it is crucial for emphasis to be placed on public service. Advertently or inadvertently focusing on financial and material benefits could be counterproductive – shortfalls in meeting can fuel perceptions of maleficence and consequently, partners dropping out. Although incentives to the private sector have been found to be relevant for improved TB control efforts (31), it is equally workable without incentives (32). As we found, some of the facilities considered TB control as a public health necessity. What this foretells for policy and planning is the importance of highlighting on public service as some respondents revealed.

Our respondents were also concerned about the equity of Ghana’s PPM for TB control. The general view was that the process of allocating TB-related infrastructure and training of personnel was inequitable. Both sectors require more continuous professional training (10, 33) as it enhances quality of TB diagnosis (10, 34). It appeared that there was lack of clarity on allocation of resources in the partnership. In resource allocation, one of the key issues that have come up is openness (35), especially when it concerns individual or institutional well-being. To cure such misconceptions of unfairness, there is a need for revisions/appeals to exist in partnerships to allow aggrieved parties to challenge and revise decisions (35).

On the issue of autonomy, from hindsight, it appears that consultation and agreements with some of the private facilities were not adequately established. Engel and van Lente (35) have made similar observations in respect of PPM in India’s TB control, where there were problems in terms of control practices and standardisation. These were sometimes accounted for by differences in organisational cultures, which were inconsistent with managerial issues in the public sector. Clashes of such nature, however minimal, could be perceived as intrusive as we noticed in this study (35). Making PPM work requires conscious efforts towards closing or minimising the internal organisational differences between the public and private partners.

The strength of this study was the inclusion of both management and frontline health personnel from both the public and private sectors. This allowed us to gain insights into the managerial and operational issues facing PPM DOTS in Ghana. Nonetheless, the present study principally focused only on the views of frontline and management of health providers, both within the public and private sectors. The findings therefore do not conclusively represent the views of all stakeholders in PPM DOTS. Certain important actors such as patients were not included in this study. Scholarship on PPM can further be enhanced with studies that combine the views of health service users and providers. Also, complimenting some of the claims/perceptions of the respondents with programmatic data would have been ideal. However, the programme was yet to set up a disaggregated data capturing system at the time of the study.

## Conclusions

Despite that all respondents acclaimed the potential benefits which can be derived from PPM DOTS, there were some implementation issues which seemed unclear at the time it was rolled out. This partly affected the smooth implementation of PPM for TB in the country. It is proposed that more attention be placed on non-maleficence, equity, and autonomy, which can invariably smoothen beneficence.

## Footnotes

1 The enablers package is a kind of stipend given to TB patients and treatment supporters to facilitate transportation, food supplements, telephone call vouchers etc. as a means of increasing treatment compliance.
